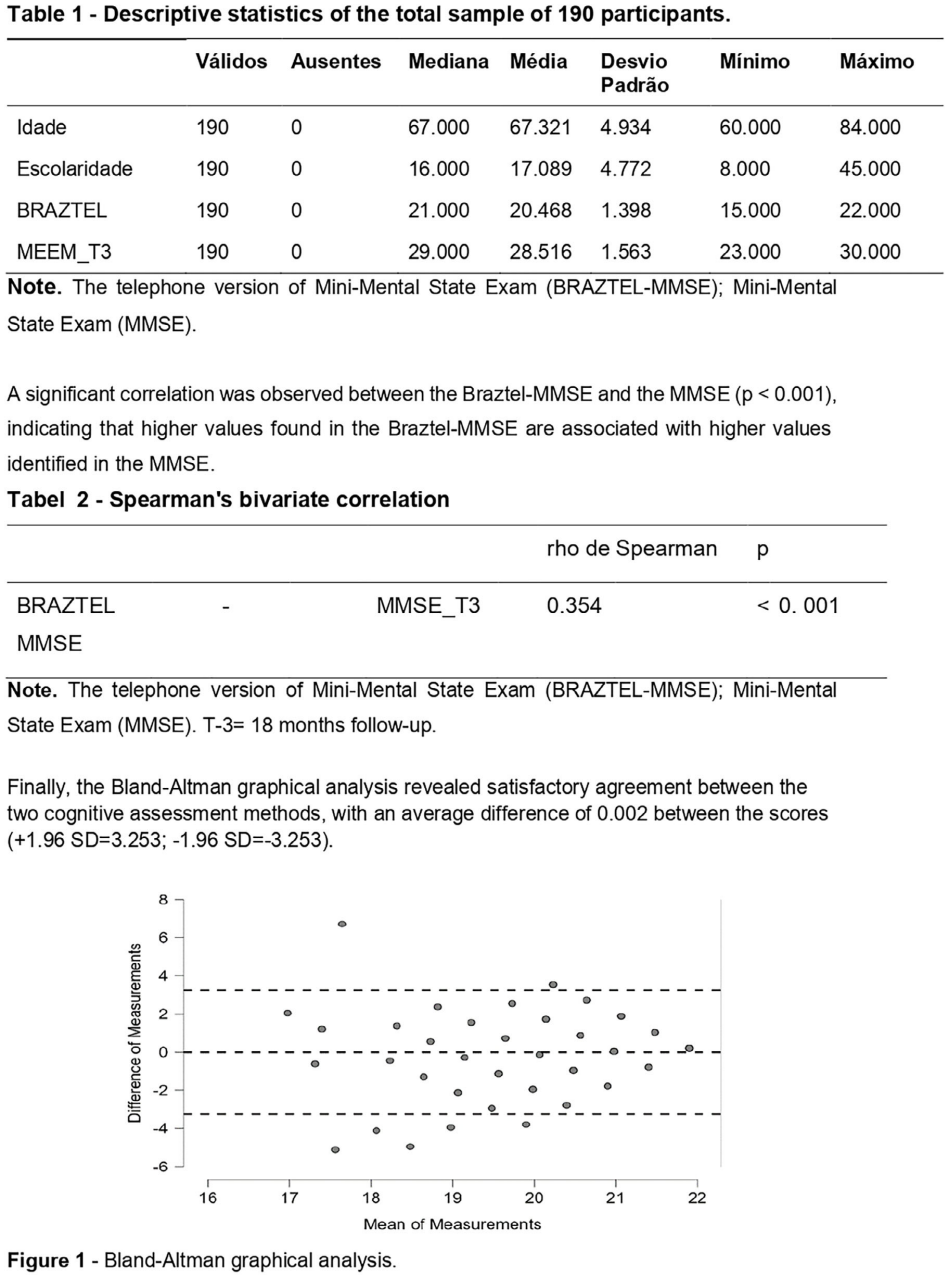# Mini‐mental state exam for telephone aplication in high‐educated socially‐engaged older adults: A descriptive study

**DOI:** 10.1002/alz70857_102046

**Published:** 2025-12-24

**Authors:** Edna Letícia de Queiroz Duarte, Gabriela dos Santos, Tiago Nascimento Ordonez, Sabrina Aparecida Da Silva, Laydiane Alves Costa, Ana Paula Bagli Moreira, Maria Antônia Antunes Fernandes, Diana Dos Santos Bacelar, Gabriela Cristina Siqueira, Patrícia Prata Lessa, Neide Pereira Cardoso, Luiz Carlos Moraes, Sonia Maria Dozzi Brucki, Thais Bento Lima Silva

**Affiliations:** ^1^ Faculty of Medicine of the University of Santo Amaro, São Paulo, São Paulo, Brazil; ^2^ Universidade de São Paulo, São Paulo, São Paulo, Brazil; ^3^ Gerontology of the School of Arts, Science and Humanities of the University of São Paulo, São Paulo, São Paulo, Brazil; ^4^ USP, São Paulo, São Paulo, Brazil; ^5^ Supera Institute of Education, São José dos Campos, São Paulo, Brazil; ^6^ Cognitive and Behavioural Neurology Unit, Hospital das Clínicas, University of São Paulo, São Paulo, São Paulo, Brazil; ^7^ Cognitive and Behavioral Neurology Group of the University of São Paulo School of Medicine, São Paulo, São Paulo, Brazil; ^8^ University of São Paulo, São Paulo, São Paulo, Brazil

## Abstract

**Background:**

The aging of the population, accompanied by growing concerns about cognitive decline and dementia, highlights the need for early accessible diagnosis. In this context, a version of the Mini‐Mental State Exam (MMSE) adapted for mobile devices and use in high‐educated socially‐engaged older adults is warranted. The effectiveness of the MMSE as a screening tool is highlighted, together with the potential of telephone‐based cognitive screening instruments, such as the telephone version of Mini‐Mental State Exam (BRAZTEL‐MMSE), as an effective accessible means of reaching older populations.

**Method:**

A descriptive study of an initial sample of 578 randomized older adult participants in a clinical trial was conducted. A subsample of 190 participants was selected from the group after 18 months to undergo the traditional MMSE. Sociodemographic information was collected for these individuals, all of whom underwent both the BRAZTEL‐MMSE by telephone and the traditional MMSE in person. Statistical analysis of the data was performed, including analysis of the correlation between scores obtained on the two instruments.

**Result:**

A significant correlation was found between scores on the BRAZTEL‐MMSE and the traditional MMSE (Spearman´s rho = 0.354, *p* < 0.001). Bland‐Altman analysis showed satisfactory agreement between the two cognitive assessment methods, with a mean difference of 8.047 points between scores.

**Conclusion:**

The study found good agreement between the BRAZTEL‐MMSE applied by telephone and the traditional Mini‐Mental State Exam (MMSE) applied face‐to‐face in an assessment of cognitive performance of older adults. These results suggest the BRAZTEL‐MMSE has potential as a useful valid tool for cognitive screening in contexts where face‐to‐face assessment is impractical, including for participants of long‐term clinical trials.